# Reproductive factors and brain volume in community-dwelling individuals: the Ohasama Study

**DOI:** 10.1265/ehpm.25-00400

**Published:** 2026-08-07

**Authors:** Ayana Saito, Kyoko Nomura, Michihiro Satoh, Satoshi Yamaguchi, Azusa Hara, Melanie Griffith-Quintyne, Hiroaki Shimizu, Shingo Nakayama, Megumi Tsubota-Utsugi, Takahisa Murakami, Yukako Tatsumi, Takuo Hirose, Tomoko Totsune, Naoko Mori, Kei Asayama, Masahiro Kikuya, Hirohito Metoki, Atsushi Hozawa, Takayoshi Ohkubo

**Affiliations:** 1 Department of Neurosurgery, Akita University Graduate School of Medicine, Akita, Japan; 2 Department of Environmental Health Science and Public Health, Akita University Graduate School of Medicine, Akita, Japan; 3 Department of Hygiene and Public Health, Teikyo University School of Medicine, Tokyo, Japan; 4 Division of Public Health, Hygiene and Epidemiology, Faculty of Medicine, Tohoku Medical and Pharmaceutical University, Sendai, Japan; 5 Department of Preventive Medicine and Epidemiology, Tohoku Medical Megabank Organization, Tohoku University, Sendai, Japan; 6 Division of Aging and Geriatric Dentistry, Department of Rehabilitation Dentistry, Tohoku University Graduate School of Dentistry, Sendai, Japan; 7 Laboratory of Social Pharmacy and Epidemiology, Showa Pharmaceutical University, Tokyo, Japan; 8 Akita Prefectural Hospital Organization, Akita, Japan; 9 Department of Pathology and Laboratory Medicine, Cedars-Sinai Medical Center, Los Angeles, CA, USA; 10 Division of Nephrology and Hypertension, Faculty of Medicine, Tohoku Medical and Pharmaceutical University, Sendai, Japan; 11 Division of Integrative Renal Replacement Therapy, Faculty of Medicine, Tohoku Medical and Pharmaceutical University, Sendai, Japan; 12 Department of Aging Research and Geriatric Medicine, Institute of Development, Aging and Cancer, Tohoku University, Sendai, Japan; 13 Department of Radiology, Akita University Graduate School of Medicine, Akita, Japan; 14 Tohoku Institute for Management of Blood Pressure, Sendai, Japan; 15 Division of Epidemiology, School of Public Health, Tohoku University Graduate School of Medicine, Sendai, Japan

**Keywords:** Brain volume, Reproductive factors, Women’s Health

## Abstract

**Background:**

Reproductive history can be conceptualized as a life-course exposure integrating biological and social factors. Although reproductive factors have been associated with stroke risk and cognitive function, their relationships with structural brain changes remain unclear. Evidence from general population–based studies linking reproductive factors to brain volume, an intermediate marker of cognitive decline, is limited. This study examined whether age at menarche, age at menopause, estrogen exposure duration, and parity are associated with brain volume in postmenopausal women.

**Methods:**

This cross-sectional study included 361 postmenopausal women aged ≥55 years from the Ohasama Study in Japan. Reproductive factors were obtained from a 1998 questionnaire were categorized into quartiles, and MRI data from 2009 to 2019 were analyzed with voxel-based morphometry. Associations were assessed using analysis of covariance adjusted for intracranial volume, age, birth cohort, and BMI. Effect sizes were reported as percent change and standardized effect sizes, with the smallest effect size of interest (SESOI) based on one-year age-related brain volume change.

**Results:**

After covariate adjustment, most associations between reproductive factors and brain volume were attenuated. Earlier age at menarche showed a weak trend toward larger temporal lobe volume (β = 0.59 cm^3^, 95% CI −0.03 to 1.21; p = 0.06), corresponding to a 0.63% change and 0.06 SD, which was comparable to the SESOI (±0.54% or 0.06 SD). Earlier age at menopause was associated with smaller occipital lobe volume (β = −0.52 cm^3^, 95% CI −1.01 to −0.03; p = 0.04), corresponding to a −1.08% change and 0.10 SD, exceeding the primary SESOI threshold (±0.64% or 0.06 SD), although confidence intervals included effects smaller than the SESOI. No significant associations were found for estrogen exposure or parity.

**Conclusion:**

Reproductive factors were not associated with moderate or large differences in brain volume in postmenopausal women. The slight, region-specific differences observed were comparable in magnitude to short-term age-related changes. These findings suggest that the clinical significance at the individual level is likely limited. However, subtle associations at the population level cannot be entirely excluded.

**Supplementary information:**

The online version contains supplementary material available at https://doi.org/10.1265/ehpm.25-00400.

## Graphical Abstract

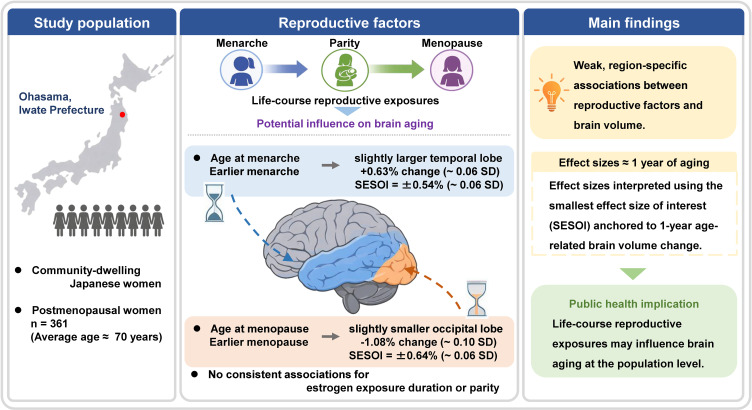

## Introduction

From a preventive medicine perspective, reproductive history can be conceptualized as a life-course exposure that integrates biological, hormonal, and social factors experienced across the lifespan. Previous studies have demonstrated associations between reproductive factors and stroke risk [1, 2]. We have previously reported significant associations between parity and silent cerebrovascular diseases (SCDs), defined as asymptomatic cerebrovascular lesions and carotid atherosclerosis, as well as cognitive function among elderly Japanese women residing in rural areas [3, 4]. Furthermore, even after adjusting for atherosclerotic lesions as mediating factors, the risk of cognitive decline remained unchanged, suggesting that atherosclerosis may not play a major role in the mechanisms linking parity and cognitive decline. These findings indicate that factors other than atherosclerosis may influence brain structure and function at stages preceding the clinical onset of stroke or dementia. If atherosclerosis plays only a limited role in its effects on cognitive function, sex hormones such as estrogen may be involved as alternative or complementary influencing factors. Estrogen has been shown to exert neuroprotective effects by enhancing synaptic plasticity, suppressing inflammation, reducing amyloid-β accumulation, and inhibiting tau hyperphosphorylation [5–7], and has been implicated in biological pathways related to the development and progression of dementia [8].

**Fig. 1:**
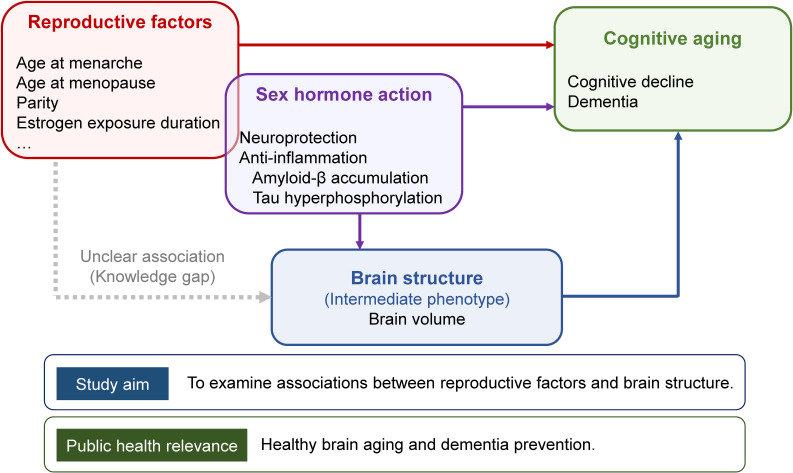
Conceptual figure.

Brain volume is known to decline with aging [9], and gray matter volume has also been proposed as a predictor of cognitive function [10]. However, evidence regarding the effects of reproductive factors on brain structure has been inconsistent, and data from general population–based studies remain limited [11–15]. Identifying intermediate phenotypes that reflect the cumulative impact of lifelong exposures and link long-term exposures to future cognitive decline is essential for early risk stratification and the development of population-based prevention strategies. In particular, studies focusing on community-dwelling populations may provide valuable insights into the primary prevention of age-related cognitive disorders in the general population (Fig. 1).

In this study, we examined whether reproductive factors, including age at menarche, age at menopause, estrogen exposure duration, and parity, are associated with MRI-derived brain volumes among postmenopausal women residing in rural areas. Because brain volume is strongly influenced by aging, we additionally examined age-related changes in brain volume within strata defined by reproductive factors to contextualize the magnitude of observed associations.

## Methods

### Design

This study employed a cross-sectional design with retrospectively collected exposure data, and was part of the Ohasama Study, a community-based longitudinal cohort in Ohasama, Iwate Prefecture, Japan, as described previously [16, 17]. The health screening program began in 1986, and participants have been continuously followed since then. In 1998, a self-administered questionnaire was used to retrospectively collect reproductive factors (age at menarche, age at menopause, and parity). Carotid ultrasonography and brain MRI have been conducted at four-year intervals. For the present analysis, we used MRI data obtained between 2009 and 2019 (after scanner upgrade) and carotid ultrasonography data obtained between 1992 and 2018, targeting women aged ≥55 years as of 1998.

### Study population

In 2010, the total population aged ≥55 years was 3426, including 1,809 women. Among 453 women who underwent brain MRI between 2009 and 2019, we excluded those whose MRI data were acquired but unavailable for analysis (n = 4), those with poor image quality (n = 64), those with infarction, intracranial hemorrhage, or mass lesions (n = 3), and those with severe white matter abnormalities (n = 4). This resulted in 378 women with analyzable MRI data. After further excluding participants with invalid or missing responses in the 1998 baseline questionnaire (n = 16) and those aged ≤54 years at MRI acquisition (n = 1), 361 women were included in the analysis.

## Reproductive factors and analytical samples

Reproductive history variables were obtained from a self-administered questionnaire conducted in 1998 and included age at menarche, age at menopause, estrogen exposure duration (defined as the interval between age at menarche and age at menopause), and parity. Because the availability of these variables differed across participants, variable-specific analytical samples were constructed using a complete-case approach, resulting in overlapping but non-identical study populations.

**Fig. 2:**
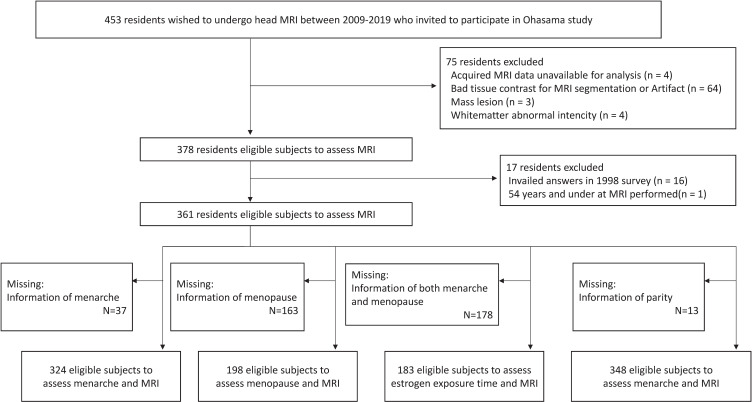
Participants’ enrollment flowchart. Variable-specific analytical samples were constructed using a complete-case approach based on the availability of reproductive history data from the 1998 questionnaire, resulting in overlapping but non-identical study populations. Missing values reflect unavailable or non-response data.

Among the 361 eligible women, analyses included 324 women for age at menarche, 198 for age at menopause, 183 for estrogen exposure duration, and 348 for parity, after exclusion of participants with missing data for the corresponding variables. Accordingly, the same individuals could contribute to multiple analyses depending on data availability (Fig. 2).

Missing values reflect unavailable or non-response data in the 1998 questionnaire, particularly for menopause-related variables. Missing values for smoking status and alcohol consumption similarly reflect non-response to lifestyle-related questions.

### Reproductive factors

Reproductive factors were collected in 1998. Age at menarche was categorized into quartiles as ≤12, 13, 14, and ≥15 years old; age at menopause into quartiles as ≤47, 48–49, 50–51, and ≥52 years old. Estrogen exposure duration, calculated as menopause age minus menarche age, was divided into quartiles of ≤33, 34–35, 36–37, and ≥38 years. Parity, including stillbirths, was also categorized into quartiles as 0–1, 2, 3, and ≥4 times.

### MRI and brain volume

All MRI scans were performed using the EXCELART Vantage (1.5T) system (Toshiba Medical Systems, Tochigi, Japan) installed at Hanamaki General Hospital (Iwate Prefecture, Japan).

The imaging parameters for the axial 3D gradient-echo sequence (FE3D sequence) were as follows: repetition time = 14 ms; echo time = 5.5 ms; flip angle = 20°; 110 slices; slice thickness = 1.5 mm; matrix = 256 × 256; field of view = 220 mm; pixel size = 0.8594 × 0.8594 mm; and scan time = 4 minutes and 59 seconds.

MRI data were processed using MATLAB R2019b (MathWorks, Natick, MA, USA), SPM12 (Wellcome Trust Centre for Neuroimaging, London, UK), and the WFU PickAtlas (Wake Forest University, Winston-Salem, NC, USA) [18, 19]. ROIs were defined for the frontal, temporal, parietal, occipital lobes, and cerebellum using the TD lobes atlas [20], and for the hippocampus and amygdala using the Automated Anatomical Labeling atlas [21].

### Carotid ultrasonography

Carotid ultrasonography was performed using a real-time B-mode ultrasound unit (7.5 MHz, Sonolayer SSA-250A, Toshiba, Tokyo, Japan) by trained physicians following a standardized protocol [22]. Mean intima-media thickness (IMT) was defined as the average of the maximum near- and far-wall thicknesses of the common carotid artery. Carotid plaques were assessed bilaterally at the common, bifurcation, internal, and external carotid arteries [3].

### Covariates

Covariates included age at MRI (continuous), birth cohort (≤1943 vs >1943), education (≤junior high school vs ≥high school), Body mass index (BMI) (continuous, kg/m^2^), and smoking and alcohol consumption (ever vs never). Hypertension was defined as the use of antihypertensive medication or a mean morning home blood pressure ≥ 135/85 mmHg [23]. Diabetes mellitus was defined as fasting glucose ≥ 126 mg/dL, 2-hour glucose ≥ 200 mg/dL after a 75 g OGTT, random glucose ≥ 200 mg/dL, HbA1c ≥ 6.5%, or use of antidiabetic medication [24]. Hypercholesterolemia was defined as total cholesterol ≥ 200 mg/dL or use of lipid-lowering medication [25].

### Statistical analyses

Categorical variables were summarized as counts and percentages and compared using the chi-square or Fisher’s exact test, as appropriate. Continuous variables were expressed as means and standard deviations (SD) or medians and interquartile ranges, and compared using Welch’s t-test or the Mann–Whitney U test. For comparisons across more than three groups, one-way ANOVA or the Kruskal–Wallis test was applied.

Associations between reproductive factors and brain volumes were examined using separate ANCOVA models for each brain region, treating brain volume as a continuous dependent variable. Reproductive factors were modeled as categorical exposures, and adjustment sets were defined a priori based on directed acyclic graph (DAG). Intracranial volume (ICV) and age at MRI were included in all models. For analyses of age at menarche, estrogen exposure duration, and parity, models additionally adjusted for birth cohort to account for secular trends. For trend analyses, quartile categories were modeled as ordinal variables. Adult BMI, smoking, alcohol use, and parity were considered potential mediators and excluded from the primary models. For age at menopause, adult BMI was included as a confounder, while birth cohort was excluded to avoid overadjustment due to strong collinearity with age at menopause.

Least-squares means (marginal means) for each reproductive factor category were estimated from the adjusted models, with pairwise comparisons conducted using Bonferroni correction. Each brain region was analyzed separately, and no multiple-comparison correction was applied across regions. All analyses were performed using SAS version 9.4 (SAS Institute, Cary, NC, USA), and two-sided p < 0.05 was considered statistically significant.

### Sample size and precision

This study was a secondary analysis of imaging participants from an existing community-based cohort (the Ohasama Study); therefore, no a priori sample size calculation was performed. Rather than relying solely on statistical significance, we focused on estimation and precision by presenting 95% confidence intervals (95% CI) for the primary effect estimates (β). To aid interpretation of effect magnitude, β and its 95% CI were additionally expressed as (i) percent change relative to the mean regional volume and (ii) standardized effect size (β divided by the regional standard deviation).

As a descriptive reference for contextualizing observed effects, the SESOI was defined based on the estimated age-related change in brain volume over one year and expressed in the same units as the reported estimates (percent change and standardized units), following a recent recommendation to anchor effect size interpretation to contextually meaningful benchmarks rather than statistical significance alone [26]. To further examine whether estimated effects were largely contained within this range, equivalence testing using the two one-sided tests (TOST) procedure was performed as a supplementary analysis.

The SESOI framework and TOST evaluations were specified post hoc and used as exploratory tools to aid interpretation of effect magnitude rather than to define clinical or biological significance. Because multiple brain regions were examined, no familywise error correction was applied; analyses were treated as hypothesis-generating on a region-by-region basis. Percent changes, standardized effect sizes, and TOST results were derived only from the multivariable-adjusted models, which constitute the primary estimates in this study.

## Results

### Age at menarche

Among 324 women, the mean age at the time of MRI was 68.3 ± 6.9 years. 153 participants (47%) had been born before 1943 (Table 1). Participants with later menarche were older at the time of MRI (≤12 years: 64 ± 6 years; 13 years: 66 ± 6 years; 14 years: 69 ± 6 years; ≥15 years: 72 ± 6 years; *P* < 0.01) and more likely to be born before 1943 (≤12 years: 42%; 13 years: 37%; 14 years: 41%; ≥15 years: 31%; *P* < 0.01). The proportion with high school education or higher was lower among those with later menarche (≤12 years: 69%; 13 years: 53%; 14 years: 35%; ≥15 years: 26%; *P* < 0.01). Women with menarche ≥15 years had greater IMT than those with ≤12 years and 13 years group (≤12 years: 0.6 ± 0.1 mm; 13 years: 0.7 ± 0.1 mm; 14 years: 0.7 ± 0.1 mm; ≥15 years: 0.7 ± 0.1 mm; *P* < 0.01). No differences were observed in alcohol, smoking, plaques, or medical history (Supplementary Table S1).

**Table 1:** Baseline Characteristics Stratified by Reproductive Factor Groups

	**Menarche group**		**Menopause group**		**Estrogen exposure** **duration group**		**Parity group**	
	**n = 324**		**n = 198**		**n = 183**		**n = 348**	

**Variable**	**Missing, ** **n**	**mean (SD) ** **or n (%)**	**Missing, ** **n**	**mean (SD) ** **or n (%)**	**Missing, ** **n**	**mean (SD) ** **or n (%)**	**Missing, ** **n**	**mean (SD) ** **or n (%)**
ICV (cm^3^)	0	1406 (103)	0	1409 (102)	0	1410 (100)	0	1404 (105)
Age (year)	0	68 (7)	0	73 (5)	0	72 (5)	0	69 (7)
Year of birth before 1943	0	153 (47.2)	0	149 (75.3)	0	136 (74.3)	0	174 (50)
BMI (kg/m^2^)	1	23.6 (3.5)	1	23.4 (3.6)	1	23.4 (3.5)	2	23.6 (3.5)
Drinker	31	68 (23)	13	36 (20)	10	34 (20)	35	71 (23)
Smoker	31	12 (4)	13	7 (4)	10	7 (4)	35	12 (4)
High Education level	5	137 (43)	4	59 (30)	2	56 (31)	8	142 (42)
Hypertension	0	184 (57)	0	127 (64)	0	115 (63)	0	203 (58)
Hypercholesterolemia	2	195 (61)	2	115 (59)	2	109 (60)	3	206 (60)
Diabetes	2	30 (9)	2	25 (13)	2	22 (12)	2	34 (10)
IMT (mm)	4	0.7 (0.1)	3	0.7 (0.1)	2	0.7 (0.1)	6	0.7 (0.1)
Plaque	4	41 (13)	3	33 (17)	2	30 (17)	6	46 (14)

Continuous variables are presented as mean (standard deviation), and nominal variables as number (%).

In all brain regions except the frontal lobe, women with age at menarche ≥15 years tended to have the smaller brain volumes (Supplementary Table S2).

In unadjusted analyses, higher age at menarche was statistically associated with differences in regional brain volumes (all *P* < 0.05, Supplementary Table S3).

**Fig. 3:**
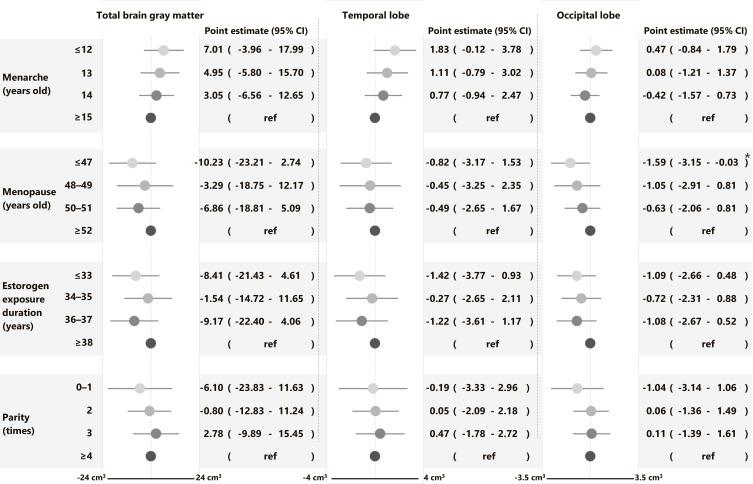
Associations between reproductive factors and total gray matter, temporal and occipital lobe volumes. Analysis of covariance examining the associations between reproductive factors and total gray matter, temporal lobe and occipital lobe volumes. Multivariable-adjusted models of age at menarche, estrogen exposure duration, and parity were adjusted for age at MRI, birth cohort (≤1943 vs >1943), and ICV. For age at menopause, multivariable-adjusted models were adjusted ICV and age at MRI and BMI. *P < 0.05.

After ANCOVA adjustment for covariates, earlier menarche was associated with a tendency toward larger temporal lobe volume (Fig. 3), however, the estimates were imprecise (β = 0.59 cm^3^, 95% CI −0.03 to 1.21; *P* = 0.06) (Table 2). In the menarche group, the age-related change in temporal lobe volume was approximately −0.54% per year (corresponding to about 0.06 SD per year) (Supplementary Table S4). Accordingly, the SESOI for the temporal lobe in this group was defined as ±0.54% (approximately one year of age-related change) or 0.06 SD. The observed association corresponded to a beta percent change of +0.63% (95% CI −0.04 to 1.30) and a standardized effect size of 0.06 SD. Relative to the SESOI reference range, the point estimate was approximately equivalent to a one-year age difference, whereas the 95% CI spanned both values within and beyond the SESOI bounds. In equivalence testing using the TOST procedure, the 95% CI was not entirely contained within the equivalence bounds, and therefore statistical equivalence was not demonstrated. Taken together, although the point estimate suggests an association corresponding to approximately one year of age-related difference in temporal lobe volume, the wide confidence interval, including effects smaller than the SESOI, indicates substantial uncertainty regarding the practical significance of this association.

**Table 2:** Association Between age at Menarche and Brain Volume

	**Beta**	**95% CI**	**P for trend**	**Beta percent change ** **(%)**	**Standardized effect size ** **(SD unit)**
Total brain gray matter	2.32	−1.19–5.84	0.19	0.41	0.05
Frontal lobe	0.65	−1.07–2.37	0.46	0.45	0.04
**Temporal lobe**	**0.59**	**−0.03–1.21**	**0.06**	**0.63**	**0.06**
Parietal lobe	0.22	−0.35–0.79	0.45	0.34	0.03
Occipital lobe	0.17	−0.26–0.59	0.44	0.34	0.03
Cerebellum	0.00	−0.60–0.61	0.25	0.00	0.00
Hippocampus	0.04	−0.03–0.12	0.22	0.68	0.06
Amygdala	0.01	−0.01–0.03	0.63	0.62	0.07
Total brain white matter	−0.92	−4.67–2.83	0.19	−0.21	0.02

Unit of β: cm^3^. Multivariable linear regression (ANCOVA) models were used to examine trends in brain volume associated with a decrease in age at menarche. Models were adjusted for ICV, age at MRI, and birth cohort (≤1943 vs >1943). β represents the estimated change in brain volume associated with a one-quartile decrease in age at menarche. Effect estimates are presented as (i) percentages relative to the mean regional volume and (ii) standardized effect sizes (β divided by the regional standard deviation).

### Age at menopause

Among 198 women, the mean age at the time of MRI was 72.6 ± 5.4 years. Most participants were born in or before 1943, accounting for 149 individuals (75.3%; Table 1). The proportion of those born before 1943 increased steadily with age and reached 96% in the ≥52 year group (≤47 years: 68.6%; 48–49 years: 63%; 50–51 years: 66%; ≥52 years: 96%; *P* < 0.01). No other covariates differed significantly among groups (Supplementary Table S6).

In the unadjusted analyses, no linear trend was observed across groups (Supplementary Table S3). After covariate adjustment using ANCOVA, earlier age at menopause was associated with a tendency toward smaller occipital lobe volume (β = −0.52 cm^3^, 95% CI −1.01 to −0.03; P = 0.04) (Table 3, Fig. 3). In the menopause group, the age-related change in occipital lobe volume was approximately −0.64% per year (corresponding to about 0.06 SD per year) (Supplementary Table S4). Accordingly, the SESOI for the occipital lobe in this group was defined as ±0.64% (approximately one year of age-related change) or 0.06 SD. The observed association corresponded to a beta percent change of −1.08% (95% CI −2.11 to −0.06) and a standardized effect size of 0.10 SD. Relative to the SESOI reference range, the point estimate exceeded the primary SESOI threshold and corresponded to approximately 1.7 years of age-related difference. However, the 95% confidence interval included values both exceeding and below the SESOI. In equivalence testing using the TOST procedure, the 95% CI was not fully contained within the equivalence bounds, and therefore statistical equivalence was not demonstrated. Taken together, these findings suggest an association between earlier menopause and smaller occipital lobe volume; however, the magnitude of the effect remains uncertain because the confidence interval includes effects smaller than the SESOI.

**Table 3:** Association Between age at Menopause and Brain Volume

	**Beta**	**95% CI**	**P for trend**	**Beta percent change ** **(%)**	**Standardized effect size ** **(SD unit)**
Total brain gray matter	−2.75	−6.84–1.35	0.19	**−**0.49	0.05
Frontal lobe	0.17	−1.87–2.21	0.87	0.12	0.01
Temporal lobe	−0.24	−0.98–0.50	0.52	**−**0.26	0.03
Parietal lobe	−0.49	−1.16–0.18	0.15	**−**0.77	0.07
**Occipital lobe**	**−0.52**	**−1.01**–**−0.03**	**0.04**	**−1.08**	**0.10**
Cerebellum	−0.03	−0.80–0.74	0.95	**−**0.03	0.00
Hippocampus	−0.01	−0.10–0.08	0.86	**−**0.12	0.01
Amygdala	0.01	−0.01–0.03	0.43	0.47	0.05
Total brain white matter	−0.77	−5.13–3.58	0.73	**−**0.18	0.02

Unit of Beta: cm^3^. Multivariable linear regression (ANCOVA) models were used to examine trends in brain volume associated with a decrease in age at menopause. Models were adjusted for ICV and age at MRI and BMI. β represents the estimated change in brain volume associated with a one-quartile decrease in age at menopause. Effect estimates are presented as (i) percentages relative to the mean regional volume and (ii) standardized effect sizes (β divided by the regional standard deviation).

### Estrogen exposure duration

Among 183 women, the mean age at the time of MRI was 72.2 ± 5.3 years, and 136 participants (74.3%) were born before 1943 (Table 1). No other covariates differed significantly among groups (Supplementary Table S7). Brain volumes did not differ significantly among exposure groups (Supplementary Table 2). In univariate analysis, occipital lobe volume was slightly smaller in the ≤33 years estrogen exposure duration group (*P* < 0.05) but showed no linear trend (Supplementary Table S3). In ANCOVA, no significant associations were found (Supplementary Table S5, Supplementary Fig. F1, F2). When interpreted against the SESOI defined by the age-related change in brain volume over one year, the observed differences were small and did not support a meaningful association between estrogen exposure duration and regional brain volumes.

### Parity

Among 348 women, the mean age at the time of MRI scanning was 68.8 ± 7.1 years, and 174 (50%) were born before 1943. No significant differences were seen in any covariates across parity groups (Table 1, Supplementary Table S8). No significant differences in total or regional brain volumes were found by parity (Supplementary Table S2). Neither univariate linear regression nor ANCOVA revealed significant associations between parity and any brain region (Supplementary Table S3, S5, Supplementary Fig. F1, F2). When interpreted against the SESOI defined by the age-related change in brain volume over one year, the observed differences were small and did not support a meaningful association between parity categories and regional brain volumes.

## Discussion

In this study, we examined associations between reproductive factors and brain volumes among elderly women living in a rural area of Japan. Overall, we observed weak and region-specific relationships. Earlier age at menarche was associated with a tendency toward larger temporal lobe volumes, whereas earlier age at menopause was associated with a tendency toward smaller occipital lobe volumes. Other reproductive factors showed no consistent associations with brain structure. Importantly, the magnitudes of these associations were small and should be interpreted cautiously.

To contextualize these findings, we interpreted effect estimates relative to the SESOI, defined as the age-related change in brain volume over one year. Prior studies suggest that an annual brain volume decline of approximately 0.3 to 0.5% represents normal aging in healthy populations [9]. Because this study was a secondary analysis, the SESOI was specified post hoc and used as a descriptive interpretative framework rather than a threshold for clinical or biological significance. Within this framework, our results suggest that even if associations between reproductive factors and regional brain volumes exist, their magnitudes are likely small relative to typical age-related changes, although slight small regional effects cannot be excluded.

These findings should be considered in the broader context of dementia research. Dementia prevalence is particularly high in Japan [27–29], and women are disproportionately affected. Hormonal fluctuations during the menopausal transition have been proposed as a potential contributor to sex differences in Alzheimer’s disease risk [30]. However, previous studies examining reproductive factors and brain structure have yielded inconsistent results, and evidence among Asian populations remains limited [11–15]. Our findings do not support moderate or large structural differences in brain volumes associated with reproductive factors in later life.

From a biological perspective, estrogen is known to exert neuroprotective effects [31, 32] and its receptors are widely expressed in the brain, particularly in temporal regions [33–36]. This provides a plausible context for the weak association observed between age at menarche and temporal lobe volume. Similarly, age-related vulnerability of the occipital cortex may partly explain the small association observed with age at menopause [37]. However, given the slight effect sizes observed and the advanced age of participants, these interpretations remain speculative, and any neuroprotective influence of estrogen on brain volume appears limited in later life.

Notably, no associations were observed in the hippocampus or amygdala. Although these regions are sensitive to estrogen [38], they are also highly plastic and undergo substantial age-related changes [39]. Given that many participants were decades past menopause, earlier hormonal influences may no longer be detectable as measurable volumetric differences.

An additional strength of this study is the use of an effect-size–based interpretative framework incorporating SESOI, rather than relying solely on statistical significance. This approach aligns with recent recommendations from large-scale neuroimaging studies, which emphasize estimation, effect sizes, and uncertainty over dichotomous significance testing. By adopting this framework, we aimed to avoid overinterpretation of statistically detectable but practically small effects [26].

In our previous analyses using the same cohort, higher parity was associated with asymptomatic ischemic lesions, carotid plaques, and cognitive impairment [3, 4]. In contrast, no association between parity and brain volume was observed in the present study. This discrepancy suggests that previously reported associations may reflect indirect pathways, such as vascular or metabolic mechanisms, rather than direct structural effects on the brain. Sociocultural factors may also contribute. In the examined birth cohorts, women with 0–1 child were uncommon in rural Japan, and such low-parity women may have had underlying frailty, infertility, or social disadvantage associated with higher mortality [40–42]. Accordingly, the null findings for parity and brain volume should be interpreted in the context of potential survival and selection biases.

Although reproductive factors are often discussed within the context of sex-specific medicine, the findings of this study have broader implications from the perspectives of preventive medicine and environmental health. Reproductive history reflects cumulative lifelong exposures, including hormonal changes, socioeconomic conditions, and health-related behaviors, all of which may influence brain aging processes. By examining associations between reproductive factors and brain volume, an established predictor of future cognitive decline, this study contributes to the identification of early, subclinical markers relevant to dementia prevention in the general population. From a population-health perspective, these findings provide insight into potential life-course pathways linking reproductive aging to cognitive vulnerability.

Several limitations should be noted. First, the cross-sectional design precludes causal inference. Second, participants were relatively healthy older women, and historical socioeconomic and nutritional conditions, particularly those before World War II, may have influenced both brain development and reproductive timing, introducing potential selection bias [43, 44]. Third, information on hormonal exposure was limited; data on circulating hormone levels, hormone therapy use, breastfeeding history, and oral contraceptive use were unavailable, and ages at menarche and menopause were self-reported, raising the possibility of recall bias.

Despite these limitations, this study provides MRI-based evidence from a community-dwelling population of Japanese women and contributes descriptive insights into reproductive aging and brain morphology while avoiding overinterpretation of small effect sizes.

## Conclusion

The findings of this study do not support moderate or large neuroprotective effects of lifetime endogenous estrogen exposure on brain volume. Earlier menarche showed only a weak, region-specific tendency toward larger temporal lobe volume, whereas earlier menopause was weakly associated with smaller occipital lobe volume, with slight standardized effect sizes. These observations suggest that, although slight regional differences cannot be entirely excluded, the magnitude of any association between reproductive factors and brain structure is limited. These findings underscore the importance of cautious interpretation of statistically detectable but small structural differences when considering reproductive history as a target for population-based dementia prevention.

## Abbreviations

AD: Alzheimer’s disease
ANCOVA: analysis of covariance
ANOVA: one-way analysis of variance
BMI: body mass index
CVD: cardiovascular diseases
DAG: directed acyclic graph
ICV: intracranial volume
IMT: mean intima-media thickness
MMSE: Mini-Mental State Examination
OECD: Organisation for Economic Co-operation and Development
SCDs: silent cerebrovascular diseases
SD: standard deviation
SESOI: smallest effect size of interest
TOST: two one-sided tests

## Supplementary information

**Additional file 1:** **Supplementary Table S1.** Baseline characteristics of participants according to age at menarche. **Supplementary Table S2.** Comparison of brain volumes by reproductive factor groups. **Supplementary Table S3.** Associations between reproductive factors and brain volume (univariate regression analyses). **Supplementary Table S4.** Associations between age and brain volume stratified by reproductive factor groups (multivariable-adjusted regression analyses). **Supplementary Table S5.** Associations between reproductive factors and brain volume (multivariable-adjusted regression analyses). **Supplementary Table S6.** Baseline characteristics of participants according to age at menopause. **Supplementary Table S7.** Baseline characteristics of participants according to estrogen exposure duration. **Supplementary Table S8.** Baseline characteristics of participants according to parity.

**Additional file 2:** **Supplementary Figure F1** Analysis of covariance examining the associations between reproductive factors and total brain white matter, frontal lobe and parietal lobe volumes.

**Additional file 3:** **Supplementary Figure F2** Analysis of covariance examining the associations between reproductive factors and cerebellar, hippocampal, and amygdala volumes.

## Declarations

### Availability of data and materials

Data available on request from authors.
