# Supplementary material for: Reproductive factors and brain volume in community-dwelling individuals: the Ohasama Study
Source: Environ Health Prev Med. 2026 Aug 7;31:52. doi: 10.1265/ehpm.25-00400 (PMC13482161; doi:10.1265/ehpm.25-00400)
Supplement: Supplementary file 1 [file ehpm-31-052-s002.pdf]

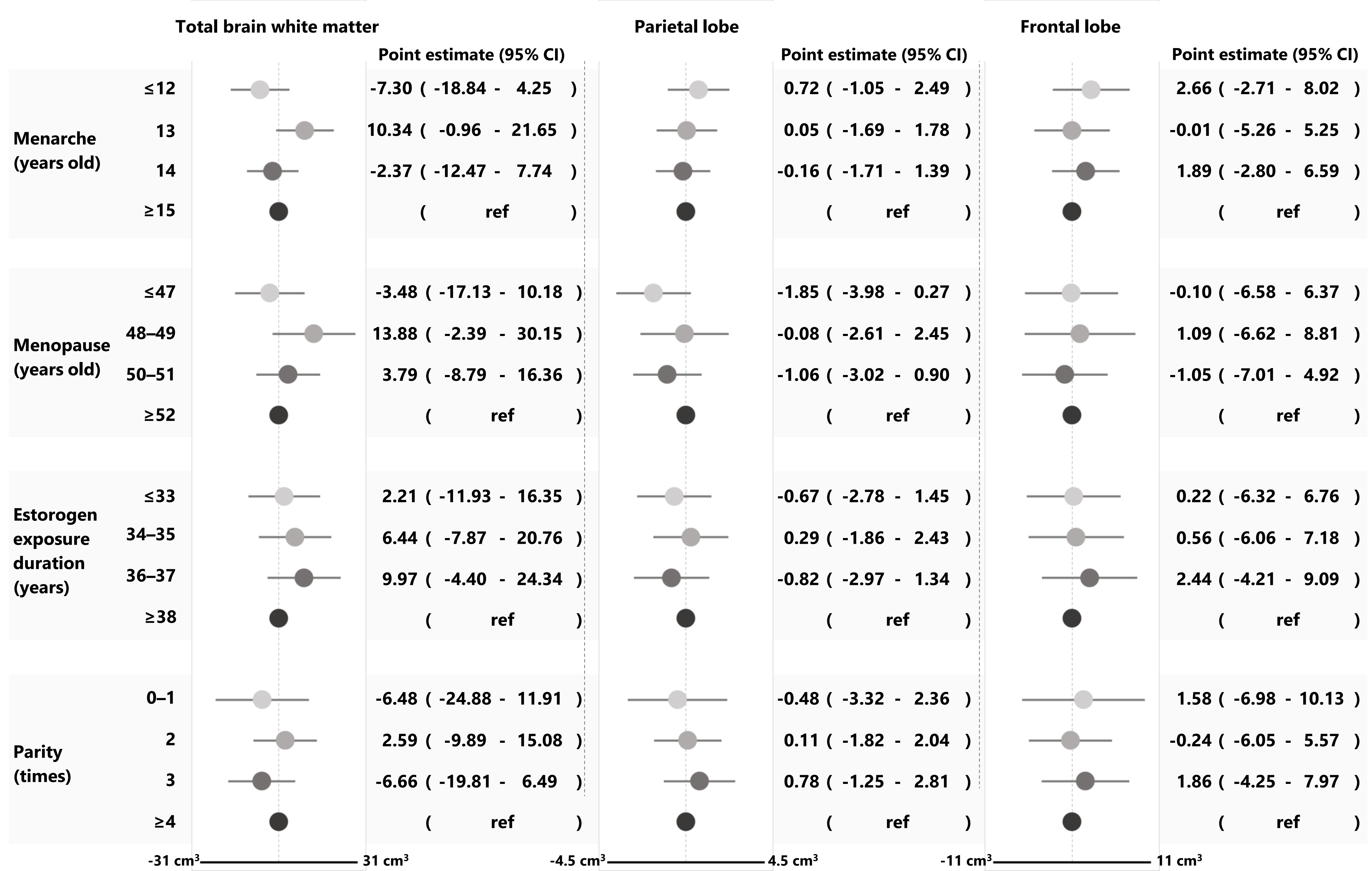

Variable	Category	Total brain white matter: Point estimate (95% CI)	Parietal lobe: Point estimate (95% CI)	Frontal lobe: Point estimate (95% CI)
Menarche (years old)	≤12	-7.30 (-18.84 - 4.25)	0.72 (-1.05 - 2.49)	2.66 (-2.71 - 8.02)
	13	10.34 (-0.96 - 21.65)	0.05 (-1.69 - 1.78)	-0.01 (-5.26 - 5.25)
	14	-2.37 (-12.47 - 7.74)	-0.16 (-1.71 - 1.39)	1.89 (-2.80 - 6.59)
	≥15	(ref)	(ref)	(ref)
Menopause (years old)	≤47	-3.48 (-17.13 - 10.18)	-1.85 (-3.98 - 0.27)	-0.10 (-6.58 - 6.37)
	48–49	13.88 (-2.39 - 30.15)	-0.08 (-2.61 - 2.45)	1.09 (-6.62 - 8.81)
	50–51	3.79 (-8.79 - 16.36)	-1.06 (-3.02 - 0.90)	-1.05 (-7.01 - 4.92)
	≥52	(ref)	(ref)	(ref)
Estorogen exposure duration (years)	≤33	2.21 (-11.93 - 16.35)	-0.67 (-2.78 - 1.45)	0.22 (-6.32 - 6.76)
	34–35	6.44 (-7.87 - 20.76)	0.29 (-1.86 - 2.43)	0.56 (-6.06 - 7.18)
	36–37	9.97 (-4.40 - 24.34)	-0.82 (-2.97 - 1.34)	2.44 (-4.21 - 9.09)
	≥38	(ref)	(ref)	(ref)
Parity (times)	0–1	-6.48 (-24.88 - 11.91)	-0.48 (-3.32 - 2.36)	1.58 (-6.98 - 10.13)
	2	2.59 (-9.89 - 15.08)	0.11 (-1.82 - 2.04)	-0.24 (-6.05 - 5.57)
	3	-6.66 (-19.81 - 6.49)	0.78 (-1.25 - 2.81)	1.86 (-4.25 - 7.97)
	≥4	(ref)	(ref)	(ref)
