# Supplementary material for: Reproductive factors and brain volume in community-dwelling individuals: the Ohasama Study
Source: Environ Health Prev Med. 2026 Aug 7;31:52. doi: 10.1265/ehpm.25-00400 (PMC13482161; doi:10.1265/ehpm.25-00400)
Supplement: Supplementary file 2 [file ehpm-31-052-s003.pdf]

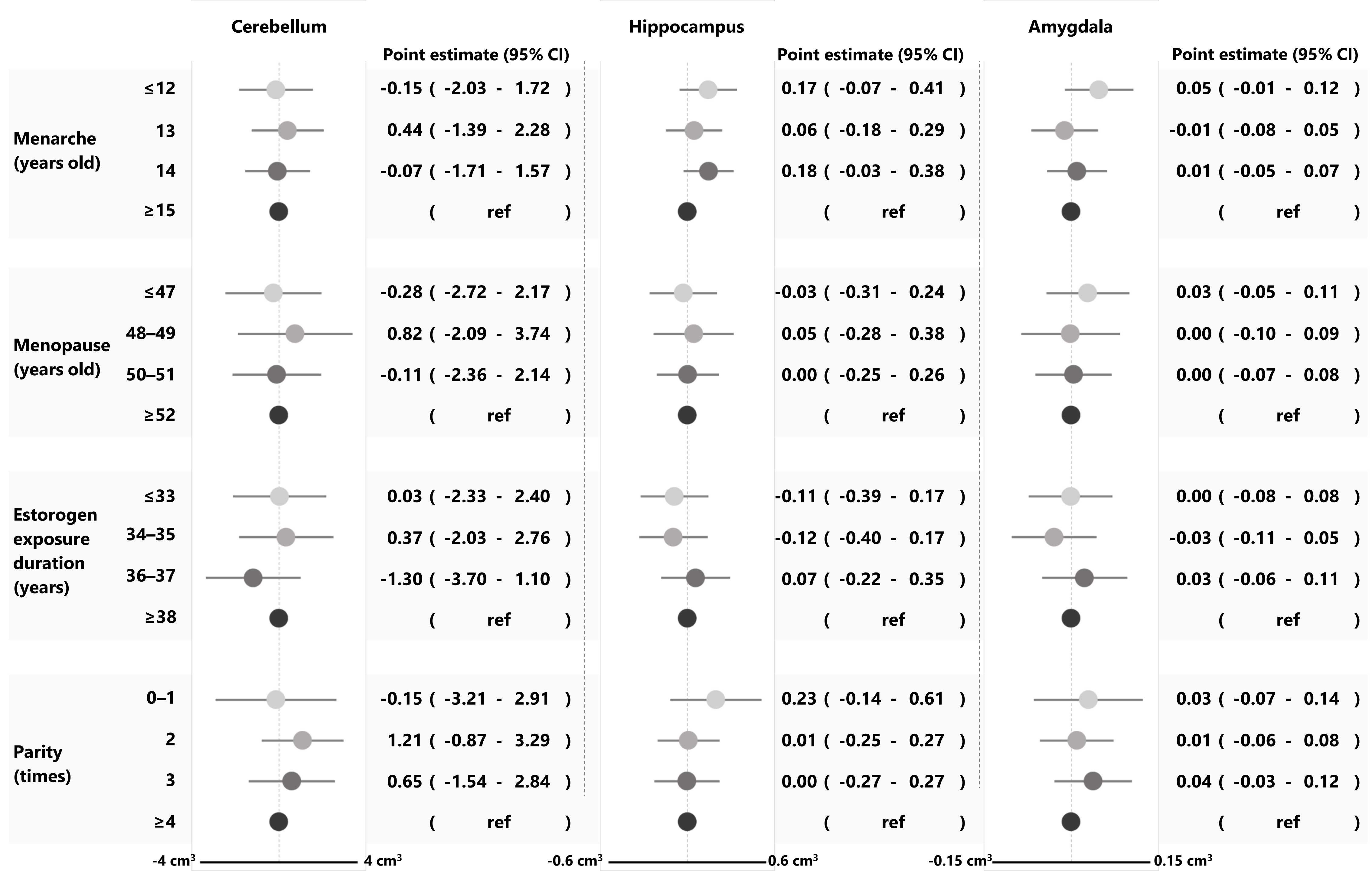

		Cerebellum Point estimate (95% CI)	Hippocampus Point estimate (95% CI)	Amygdala Point estimate (95% CI)
Menarche (years old)	≤12	-0.15 (-2.03 - 1.72)	0.17 (-0.07 - 0.41)	0.05 (-0.01 - 0.12)
	13	0.44 (-1.39 - 2.28)	0.06 (-0.18 - 0.29)	-0.01 (-0.08 - 0.05)
	14	-0.07 (-1.71 - 1.57)	0.18 (-0.03 - 0.38)	0.01 (-0.05 - 0.07)
	≥15	(ref)	(ref)	(ref)
Menopause (years old)	≤47	-0.28 (-2.72 - 2.17)	-0.03 (-0.31 - 0.24)	0.03 (-0.05 - 0.11)
	48–49	0.82 (-2.09 - 3.74)	0.05 (-0.28 - 0.38)	0.00 (-0.10 - 0.09)
	50–51	-0.11 (-2.36 - 2.14)	0.00 (-0.25 - 0.26)	0.00 (-0.07 - 0.08)
	≥52	(ref)	(ref)	(ref)
Estorogen exposure duration (years)	≤33	0.03 (-2.33 - 2.40)	-0.11 (-0.39 - 0.17)	0.00 (-0.08 - 0.08)
	34–35	0.37 (-2.03 - 2.76)	-0.12 (-0.40 - 0.17)	-0.03 (-0.11 - 0.05)
	36–37	-1.30 (-3.70 - 1.10)	0.07 (-0.22 - 0.35)	0.03 (-0.06 - 0.11)
	≥38	(ref)	(ref)	(ref)
Parity (times)	0–1	-0.15 (-3.21 - 2.91)	0.23 (-0.14 - 0.61)	0.03 (-0.07 - 0.14)
	2	1.21 (-0.87 - 3.29)	0.01 (-0.25 - 0.27)	0.01 (-0.06 - 0.08)
	3	0.65 (-1.54 - 2.84)	0.00 (-0.27 - 0.27)	0.04 (-0.03 - 0.12)
	≥4	(ref)	(ref)	(ref)
